# Main problems experienced by the neighbors of open drug scenes, Tehran, Iran: a mixed-method study

**DOI:** 10.1186/s12954-023-00880-0

**Published:** 2023-10-16

**Authors:** Sahar Eshrati, MohammadBagher Saberizafarghandi, Meroe Vameghi, Reza Arezoomandan, Hadi Ranjbar, Thomas Clausen, Helge Waal

**Affiliations:** 1 Department of Addiction, School of Behavioral Sciences and Mental Health (Tehran Institute of Psychiatry), Iran University of Medical Sciences, Shahid Mansouri St, Niayesh St, Sattarkhan Ave, Tehran, Iran; 2 Social Welfare Management Research Center, University of Social Welfare and Rehabilitation Sciences, Tehran, Iran; 3 Mental Health Research Center, School of Behavioral Sciences and Mental Health (Tehran Institute of Psychiatry), Iran University of Medical Sciences, Tehran, Iran; 4 Norwegian Center for Addiction Research (SERAF), Institution of Clinical Medicine, Faculty of Medicine, University of Oslo, Kirkveien 166, Bygg 45, 0450 Oslo, Norway

**Keywords:** Experienced problems, Open drug scene, Neighbors, And mixed-method study

## Abstract

**Background:**

Despite law enforcement and health interventions, open drug scenes have led to problems in many countries. The problems are, however, insufficiently explored. There are different types of drug scenes in Iran. This study aimed to explore the issues related to neighbors of one of the drug scenes in Tehran known as Farahzad.

**Methods:**

Data were generated via semi-structured interviews in the first step of the current mixed-method study (2020–2021). Interviewees were people who use drugs (PWUDs), residents and business owners (N = 25). In the next step, a quantitative observation was conducted for eight days. The results were analyzed using conventional content analysis and descriptive statistics.

**Results:**

The perceived problems were ambivalent attitudes about drug scene-related activities, violate of the territory of the self of the effected residents, and everyday concerns. The observation results indicated that men who use drugs are involved in drug scene-related activities more than women are. PWUDs try to hide their activities from the public view. Their efforts were considered “self-regulatory strategies” in the drug scene.

**Conclusions:**

Despite efforts of PWUDs to keep their activities invisible, drug scene-related issues are intolerable for neighbors. Neighbors and PWUDs have ambivalent attitudes. While they are concerned about the human rights of each other, drug scene-related activities have disturbed the neighbor’s daily life and economic activities. Although law enforcement and harm reduction interventions reduce some of the problems, one of the approaches should be improving the coexistence between the neighbors and the residents of the drug scene to achieve broader and more sustainable compromises.

## Background

Open drug scenes (ODSs) are places where people who use drugs (PWUDs) and drug dealers come together and publicly consume and supply drugs [1]. Problems related to drug scenes are generally shared across different settings. Associated problems are a combination of conditions, behaviors, or activities that have different characteristics, extent, and forms; and result from drug consumption, supply, or dealing. This situation can negatively impact the quality of life or make it challenging to use enjoyable spaces for local residents and neighbors [2]. The level of tolerance of drug issues is a critical element in understanding the effects of drug scenes in any society [3]. In a recent study, while one-third of residents considered their encounters with drug scenes negative, one-tenth stated their experiences positive due to the level of interaction between residents and PWUDs [1]. Therefore, the full participation of affected communities is important in navigating of challenges and developing of policies and programs [1, 4, 5].

The most serious experienced problems related to ODSs are visibly dealing and consuming drugs in public, unusual and unwanted behaviors of hotspot actors, accumulation of garbage and injecting equipment in the environment, attracting drug users from other parts of the city, lingering of homeless people and sex workers in the neighborhood, and the experience of insecurity and intimidation among local residents [2, 6, 7].

There are a variety of open drug scenes in Iran [8]. Compared to other types, the Farahzad drug scene is located in a valley and is more organized, with a specific hierarchy. The various levels of power and roles in the Farahzad drug scene (situated in the north of Tehran) include nonresident trafficker (drug scene owner), watching guard, drug dealer, sex worker, and resident and nonresident PWUDs. Since the area developed a sort of independent local rules, it is difficult to provide medical and harm reduction services in this area [8, 9].

According to the literature, different societies have different responses to ODSs. This can indicate the each society’s political, legal, and cultural approaches and the degree of tolerance of drug issues [10]. Despite various law enforcement and health measures, the problems of ODSs have continued [1]. Moreover, the level of and variations in the issues are insufficiently scientifically studied and mainly described at a common sense. Although most previous studies were from high-income countries [11], this study conducted in middle–low-income countries.

In the current study, by using quantitative and qualitative methods such as in-depth interviews and observation, we tried to get an accurate picture of the experienced drug scene-related problems that local residents do not tolerate to address the most important community-acceptable responses in the next step.

## Methods

This article originates from a study that investigated drug scene-related problems and their required interventions which was conducted between November 2020 and February 2021 using qualitative and quantitative methods.

An in-depth semi-structured interview guide was developed and progressively revised following each interview transcription and coded according to the findings of the previous interviews. Field notes of each interview were also taken. The research team categorized these codes into main themes and sub-themes, and revised them to include the emerging codes. The interviews conducted by SE, continued until data saturation occurred, and the interviews no longer revealed new codes based on the comparative method of data analysis [1, 12]. Before interviews, written informed consent was obtained for recording interviews. The duration of each interview was between 45 and 90 min. Data were analyzed using conventional content analysis and software (MAXQDA) version 10th [13].

The rigor of the data was confirmed according to Koch’s criteria, including using both interview and observation methods (triangulation), asking the participants to review the findings to confirm the accuracy of their experiences (member checking), and using peer debriefing [14]. In addition, an accurate recording of the steps and suggestion of other colleagues (cross-check codes) checked the data.

With purposive and snowball sampling, interviewees included PWUDs (N = 9), residents (N = 9), and business owners (N = 7). Participants in the study were primarily men (68%), over 40 years old with a mean age of 44.52 years, and mostly completed high school or post-high school education (60%). The inclusion criteria for PWUDs were age over 18 years, living in the area for the past month, and speaking Persian. Other participants, they should have practical experiences with drug scenes. In addition, PWUDs and other participants with had no experience in drug scene-related problems, and PWUDs who were intoxicated in a way that impeded the interview, were excluded from the study. The participants were invited with a business card, and an interview was held at a drop-in center near the drug scene. Participants received USD 15 (1,500,000 rails) as compensation for their time.

**Fig. 1:**
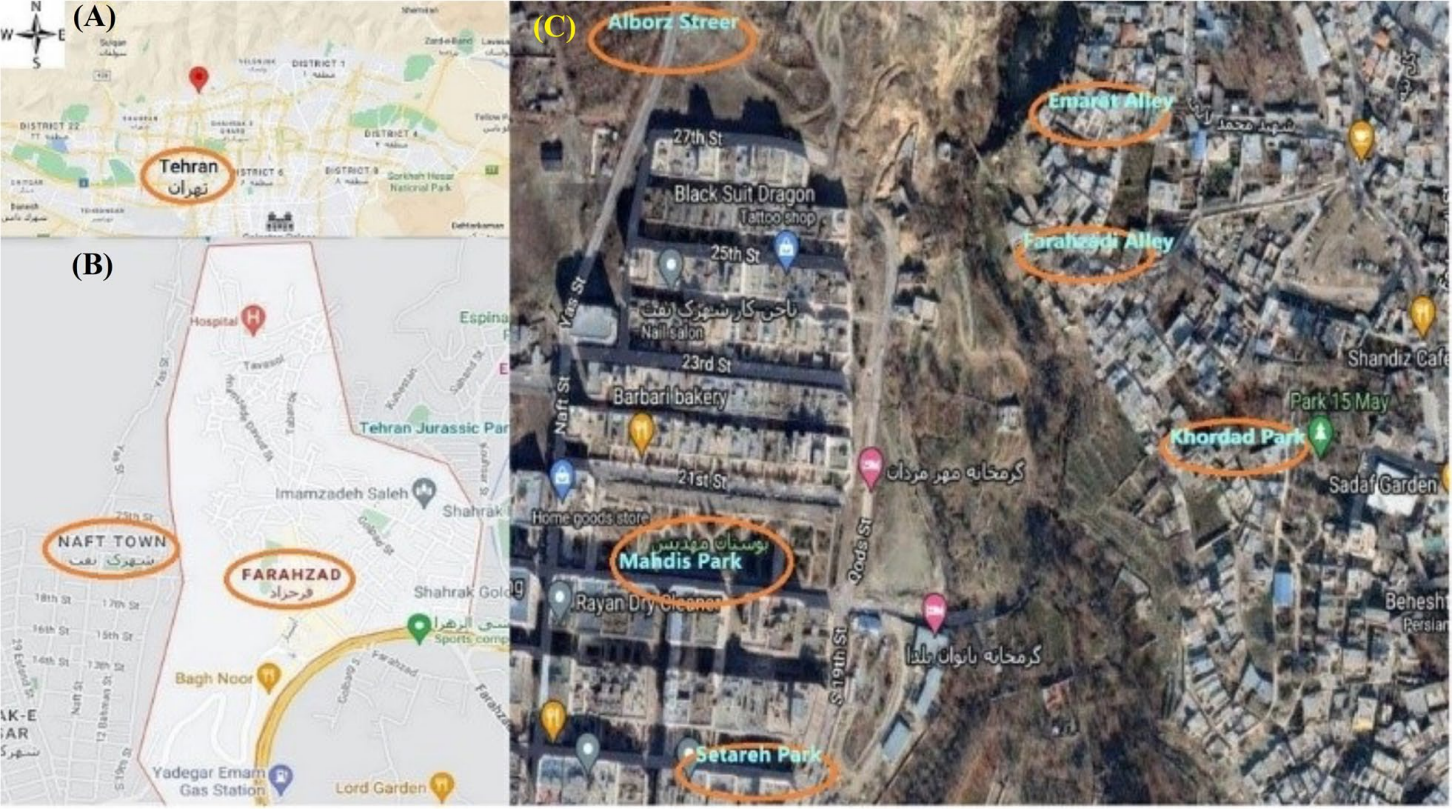
**A**, **B** Location of the Farahzad drug scene, Farahzad and Naft neighborhoods in Tehran. **C** The six locations in Farahzad and Naft neighborhoods were observed

Finally, to reinforce the qualitative findings, drug scene-related problems were observed quantitatively. An observation checklist was developed based on the qualitative findings and a previous literature review [11]. According to the suggestion of outreach staff, the police officers, the manager of the drop-in center, and local informants, the observation sites and times were selected. An observational survey was conducted eight days from 8 a.m. to 8 p.m. in six locations in Farahzad and Naft neighborhoods (Fig. 1). Farahzad valley is located between Farahzad and Naft neighborhoods. The times for observation were determined randomly so that it was possible to observe six sites at different times. To analyze the observation data, due to the limited time of the observation, we used descriptive statistics (i.e., frequency, frequency percentage, and ratio). The previous protocol study introduced detailed descriptions of methods [7].

## Results

1. Qualitative results: the experienced drug scene-related problems by local residents, business owners, and PWUDs

In-depth interviews revealed insights about the problems experienced for drug scenes by local home/business owners. Important themes emerged including (a) ambivalent attitudes about drug scene-related activities (b) violate the territory of the self of the affected residents and (c) perceived everyday concerns (Table 1).

**Table 1:** Experienced drug scene-related problems by local residents, business operators, and PWUDs

Themes	Sub-themes
Ambivalent attitudes about drug scene-related activities	Human rights versus inconvenient situations
	Obligatory versus unusual and disorder-based source of income generation
Violate the territory of the self of the affected resident	Pulling effect and exposure to public drug dealing/ using
	Exposure to drug-related paraphernalia in the public areas
	Exposure to noise and socializing of PWUDs
	Smoke from lighting fire around inhabited areas by PWUDs
Perceived everyday concerns	Housing prices and liquidity
	Not in my backyard (NIMBY) issue
	Fear of being attacked and kidnapped by PWUDs
	Relatives negative perception of neighborhood

### Ambivalent attitudes about drug scene-related activities

On the one hand, local residents felt frustrated due to encountering poor conditions of PWUDs. They want to remove drug scenes from their neighborhood. On the other hand, they acknowledge the human rights of PWUDs.

*Resident: The worst effect of the hangout for myself, who lives here, is to see these poor and miserable people, it has a harmful effect on a person's spirit, their situations, their lives, they all come to say, for example, do you buy food for me? Do you buy bread for me? Sometimes, police officers maltreat them. We want to stop patogh [hangout], but we do not want to hurt addicts like this.*

PWUDs affirm the human rights of local residents. While PWUDs believe that local residents do not deserve to be exposed drug scene-related activities, they do not have alternative places to settle down.

*PWUDs: The government should determine a place where addicts can go directly there to buy and consume drugs. It should also define some restrictions for that. For example, an addict does not have the right to take drugs out of that place. He/she should not use the drugs anywhere else. In this way, the rights of the neighbors are also respected.*

PWUDs obtain illegal drugs by engagement in disordered income generating activities such as panhandling, sex work trade, and scavenge. While affected local residents and business representative consider these activities inconvenient, they acknowledge the limited legal income generation opportunities available to PWUDs.

*Residents: The unsanitary condition of the neighborhood is related to the activities of addicts. Every addict has a bag full of garbage. They empty the contents of the trash into the bag. They carry garbage bags to the entrance of the houses. They pick up some recyclable waste, and leave the rest there. They will sell the recyclable waste to buy drugs or their daily needs.*

While PWUDs acknowledge the negative reaction of the society to their appearance due to their source of income such as gathering garbage as well as their everyday lifestyle in drug scenes, they do not have another choice.

*PWUDs: You know the way we put clothes or we gather the garbage and live in hangouts, lead to separate us from society. Out of every hundred addicts, maybe one person cares about his appearance, if he can afford it and can wear neat clothes. Otherwise, most of us may not take a shower even once a month. This way of dressing and our job makes us separated from the society. Society will never accept such people. However, with all situations, we are here and we have no choice but to scavenge to get money for drugs and our daily expenses. This is a way to earn money without worry.*

Although residents have concerns about motorcycle theft, and car break-ins, they confirmed the daily needs of PWUDs that led to the robbery.

*Resident: They stole my two motorcycles. Now you can see how I locked it, what a big chain. Most of them steal without motivation for robbery itself. They are going to earn money for their drugs, most of them are not thieves at all and have never committed theft in their lives before.*

### Violate the territory of the self of the affected resident

Some residents believed that drug dealers do not adhere the rules that normally expected from people in public spaces. Sometimes the drug dealers offered them buying drugs. Therefore, the inhabitants need to eschew them by avoiding eye contact.

*Resident: In this case, it is very catastrophizing. For example, you know that the man standing there is a drug dealer. Everyone knows that. Sometimes he approach to the passersby to sell the drugs. In this case, I need to avoid eye contact in a way that it is annoying.*

Local residents and PWUDs assumed that drug scenes made it easier for residents, especially the vulnerable neighbors and children, to initiate or escalate using drug. In addition, they believe that drug scenes attract people who use/deal drugs from all over the city.

*Resident: When my husband walks around the hangout a hundred times, he may say, “I will go and use this substance once to see what it is.”*

*PWUDs: The children see the scene. While they are watching me using drugs, they think. They remember and learn what they observe. An eleven-year-old boy is living here. He injects heroin and Shisheh [methamphetamine]. His house is in front of a Chehel Peleh [name of a hangout that means forty steps] hangout. He frequently used to see the hangout from their house’s upper floor. He says,” when I was seven, years old I wanted to see what was going on, what was the charm of it.” He was curious.*

Some drug users believed that disposal of syringes in the neighborhood near the drug scene lead to spread of infection diseases.

*PWUDs: The owners of the drug scene does not allow anyone to inject drugs. This is a rule in the drug scene. Therefore, injectors should do this elsewhere. They may be go out of the hangout and inject around the neighborhood in front of a house or in the street. At that moment, they throw their syringes there. The syringe is full of pollution, blood and so on. This could be infected.*

Some residents said that they forced to endure the noise and socializing of drug users at nights in a way that goes against normal expectations about public relation. These activities impede residents to fall sleep. In this way, noise and socializing at nights may be perceived as violation of one’s territory [11].

*Resident: We see drug users gathered. We hear their noises from the window. The window of our house overlooks this hangout. At midnight, at 12, one, two, four o'clock, drug users shout, sing, fight, and make a lot of noise. We cannot sleep. They do not listen to us. They do not calm down. We have to endure.*

Residing close to drug scenes leads to direct contact with drug scene-related activities. The material configuration of some buildings that have windows toward drug scenes can bring smoke to nearby homes due to lighting fire by PWUDs.

*Resident: Whatever they find that can burn, such as rubber, wood, plastic, whatever, paper, they fire, and they warm themselves at that moment. When the smoke rises, there is an open window [pointing to the window of their house]. All the smoke comes into the house like a chemical.*

### Perceived everyday concerns

The local home/business owners have concerns about decreasing property value (home prices and liquidity). They believed that their property’s value was adversely affected by the presence of drug scenes that almost lead to a response of “not in my backyard” (NIMBY). They have a valid concern about the presence of a drug scene, accompanied by unemployed and homeless PWUDs near the place where their home and business is located. Their perception of elevated risk in these areas could be reflected in the nearby real estate.

*Resident: here [Farahzad] is in district two; Saadat Abad [the name of another neighborhood] is also in district two. In Saadat Abad, its shops and houses are worth 200,000,000, 50,000,000 respectively. However, here its shops are worth 10,000,000 and houses are worth 3,000,000. They are in the same district. The hangouts bring it for us*.

The relatives of residents encounter various drug scene-related activities and traces when frequenting in the neighborhood. They exposure to public drug dealing/using, noise and socializing of PWUDs. They perceive them as threatening of their personal safety.

*Resident: I have many relatives and friends. In this situation, if they want to come to my house at this time of day, they call and ask me to go somewhere to accompany them. They do not dare to go to my home alone/by themselves*.

Some local residents have a fear of being attacked and kidnapped by PWUDs. They felt threatened by PWUDs. As Threadgold argues, drug scenes likely have generated a fear of deterioration of the reasonable world [15].

*Resident: A number of children disappeared in the park. For example, two small children who were 6 and 7 years old disappeared in the park suddenly. Finally, just one of them was found in the park. We do not know the details of the story. We only know that they disappeared. They are likely to have been kidnapped by addicts*.

2. Quantitative data: observations that a researcher and an outreach worker have conducted.

The findings of the observation checklist are shown in Tables 2 and 3.

**Table 2:** Frequency (frequency percentage) of drug scene-related social problems

Drug scene-related social problems	Naft neighborhood Frequency (frequency percentage %)			Farahzad neighborhood Frequency (frequency percentage %)		
	Alborz street	Mahdis park	Setareh park	15 Kordad park	Emarat alley	Farahzadi alley
Disposal syringes in public	4 (33/33)	2 (16/66)	1 (8/23)	4 (33/33)	1 (8/23)	0 (0)
Disposal foils in public	18 (11/53)	30 (19/23)	20 (12/82)	52 (33/33)	19 (12/17)	17 (10/89)
Noise						
F	0 (0)	0 (0)	0 (0)	0 (0)	0 (0)	0 (0)
M	0 (0)	0 (0)	0 (0)	1 (100)	0 (0)	0 (0)
Injection in public view (car, park, street, alley)						
F	0 (0)	0 (0)	0 (0)	0 (0)	0 (0)	0 (0)
M	0 (0)	0 (0)	0 (0)	0 (0)	0 (0)	0 (0)
Smoking in public view (car, park, street, alley)						
F	0 (0)	0 (0)	0 (0)	0 (0)	0 (0)	0 (0)
M	1 (7/14)	4 (28/57)	3 (21/42)	1 (7/14)	2 (14/28)	3 (21/42)
Intoxicated drug users						
F	0 (0)	0 (0)	0 (0)	0 (0)	0 (0)	0 (0)
M	1 (6/25)	2 (12/5)	5 (25/31)	1 (6/25)	1 (6/25)	6 (37/50)
Begging						
F	0 (0)	0 (0)	0 (0)	0 (0)	0 (0)	0 (0)
M	0 (0)	1 (50)	0 (0)	0 (0)	1 (50)	0 (0)
People who seems drug sellers						
F	0 (0)	0 (0)	0 (0)	0 (0)	0 (0)	0 (0)
M	1 (25)	0 (0)	1 (25)	1 (25)	0 (0)	1 (25)
*Loitering (standing, lingering, and walking in the neighborhood)*						
PWUDs						
F	1 (16/66)	1 (16/66)	1 (16/66)	1 (16/66)	1 (16/66)	1 (16/66)
M	0 (0)	8 (8)	20 (20)	8 (8)	18 (18)	46 (46)
Other						
F	75 (17/60)	43 (10/09)	80 (18/77)	63 (14/78)	37 (8/68)	128(30/04)
M	112(11/55)	132(13/62)	199(20/53)	114(11/76)	159(16/40)	253(26/10)
*Gathering garbage from the bin*						
PWUDs						
F	0 (0)	0 (0)	0 (0)	0 (0)	0 (0)	0 (0)
M	6 (18/18)	4 (12/12)	8 (24/24)	3 (9/09)	6 (18/18)	6 (18/18)
Other						
F	0 (0)	0 (0)	0 (0)	0 (0)	0 (0)	0 (0)
M	0 (0)	4 (44/44)	2 (22/22)	1 (11/11)	1 (11/11)	1 (11/11)
Traces of fire in neighborhood	2 (7/69)	11 (42/30)	3 (11/53)	3 (11/53)	3 (11/53)	7 (26/92)

**Table 3:** Ratio drug scene-related problems

Ratio	Alborz street (%)	Mahdis park (%)	Setareh park (%)	15 Khordad park (%)	Emarat alley (%)	Farahzadi alley (%)
Loitering of female drug users compared to male drug users	0	8	20	8	18	46
Foil disposed to that of syringes	2/5	15	20	13	19	17
Gathering of garbage by PWUDs compared to other	0	1	4	3	6	6

Females who use drugs did not participate in some drug scene-related problems such as loitering, gathering garbage, and using and dealing drugs in public. Also, both males and females who use drugs did not participate in street violence and noise, injection in public, and begging.

**Fig. 2:**
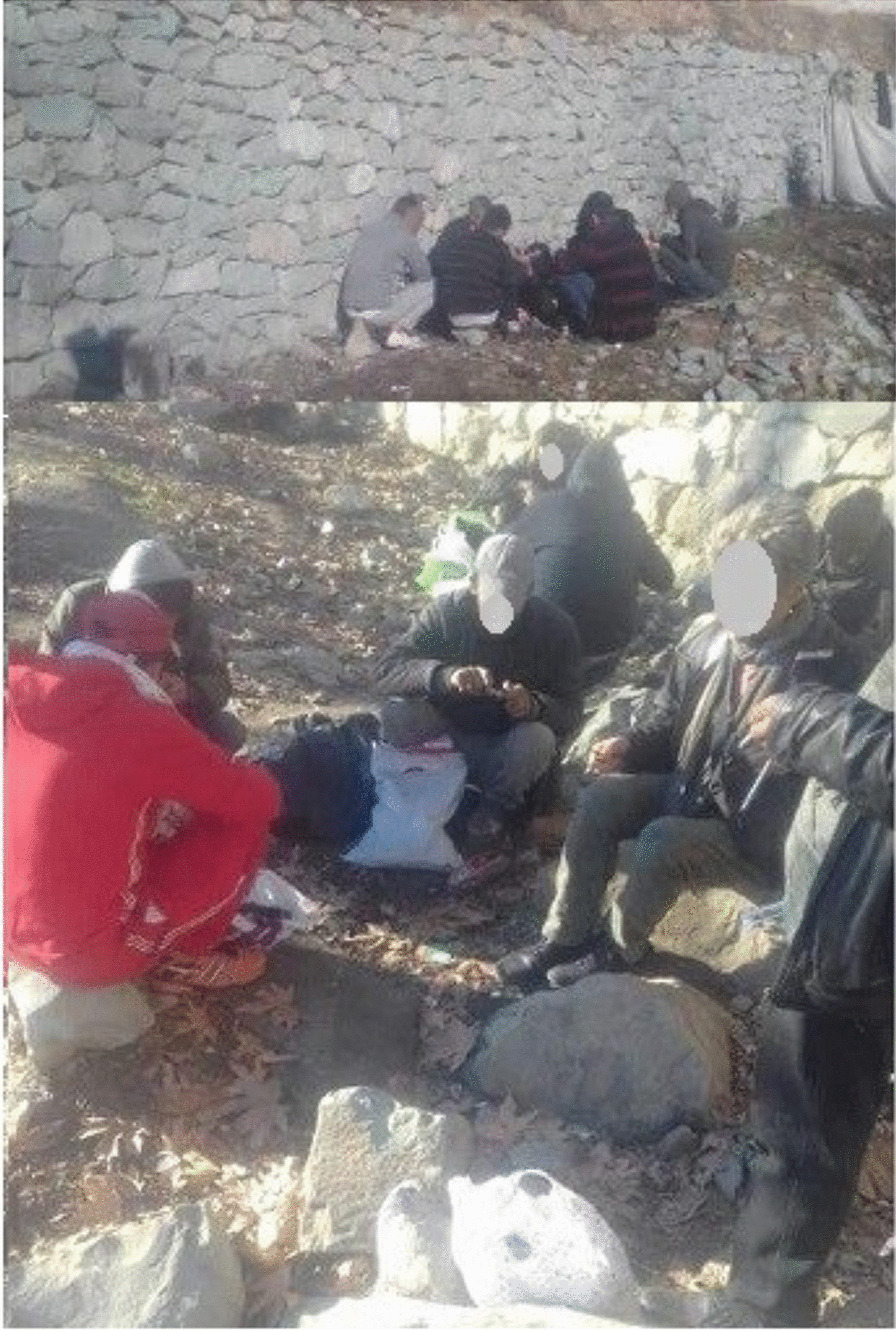
Most crowded site in Farahzadi Alley

Although the most crowded site that PWUDs was observed was in Farahzadi alley (Fig. 2), the least drug paraphernalia was abandoned in this site among other studied sites and PWUDs did not use drugs in this public area. This possibly indicates that PWUDs did not choose the crowded nearby location to take drugs. We considered these activities as “self-regulatory strategies” by PWUDs.

The ratio of drug paraphernalia disposed in the studied sites was higher than that of using drugs in public. This could possibly indicate that PWUDs used drugs more at night than during the day, possibly due to avoiding arresting by the police during the daytime.

**Fig. 3:**
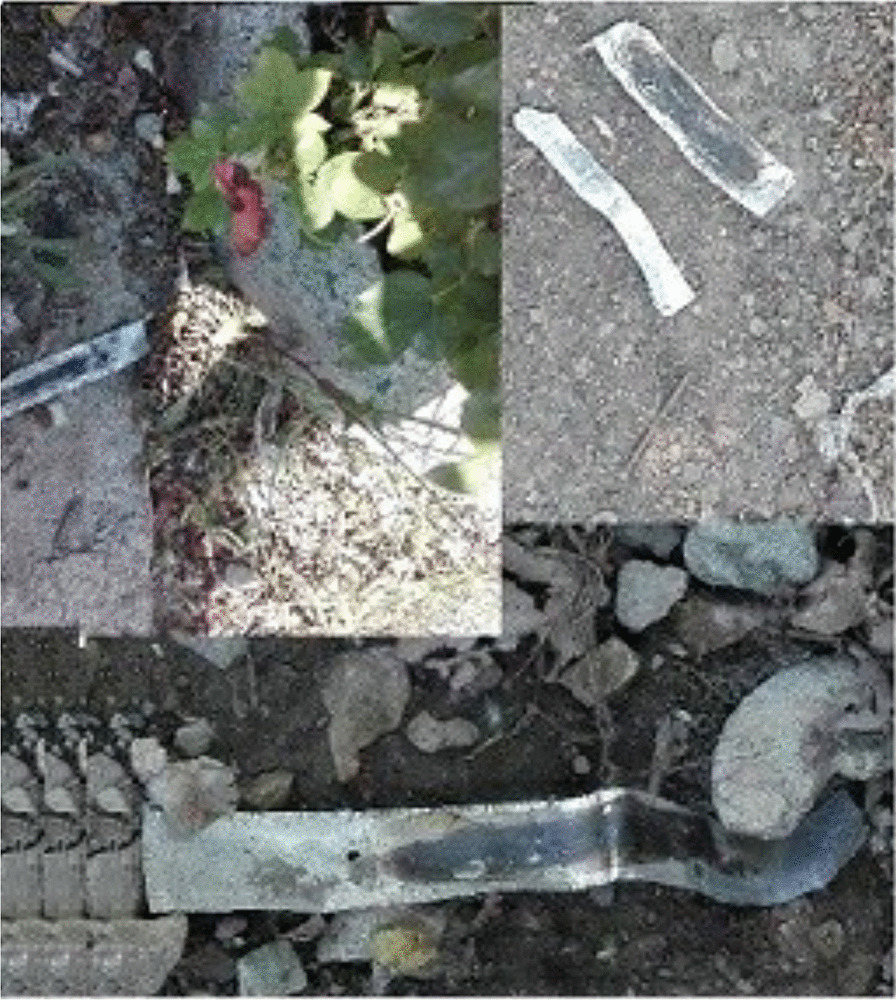
Observed disposal foils

We did not observe PWUDs injecting drugs in public view during the daytime. In addition, the ratio of foil disposed was higher than that of syringes (Fig. 3). This could be evidence the injecting of drugs at night and the success of the harm reduction innovative strategy that dig a hole to bury the syringes in the Farahzad valley. Also, smoking is the traditionally preferred method in Iran. Furthermore, drug injection did not make PWUDs high due to the quality of drugs that were distributed there.

**Fig. 4:**
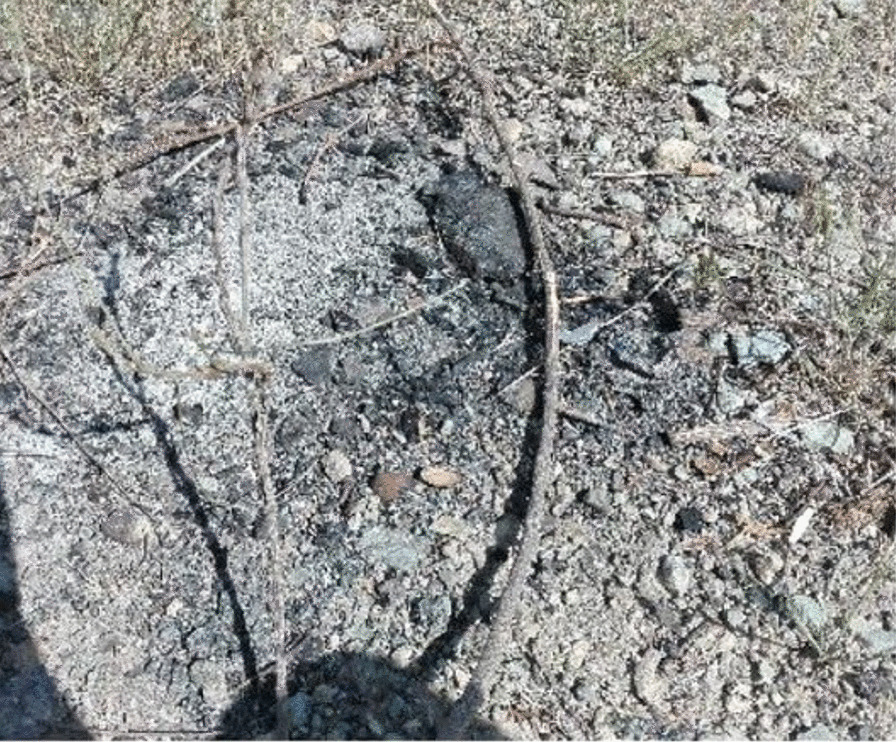
Trace of fire in the Mahdis Park

Fireplaces were more often found in Mahdis Park than in other studied sites. This might be a consequence of the urban management plan, which turned off lights in this part of the park to disperse PWUDs (Fig. 4).

As one would expect, according to the gender balance in the open drug scenes activities [16], male drug users caused more loitering than women. PWUDs gathered more garbage than other local residents indicating a source of income among PWUDs [17].

## Discussion

Although, the residents did not feel convenient from drug scene-related activities, they confirm human rights of actors of drug scenes. The results of this study suggest key contextual data regarding the experiences of PWUDs and other local residents about primary perceived drug scene-related problems, self-regulatory strategies, as well as possible interventions.

The primary drug scene-related problems that residents did not tolerate were: the attraction of PWUDs and dealers, fear of being attacked by PWUDs, exposure to drug deal and use, disposal of drug paraphernalia, noise, gathering PWUDs, smoke due to lighting fire, and decreased property values. The local affected community not only does not tolerate drug scene-related problems but also some conventional law enforcement measures such as arresting PWUDs in a discriminatory way. Public perception of the adverse effects of ODSs plays a crucial role in police operations [18]. Despite moderate benefits of the crackdown plan, these measurements are associated with violence and misconduct and negatively impact minority groups who live in the vicinity of drug scenes [19, 20].

Studies illustrate the ambiguities and mixed opinions of the public and communities about drug scene issues. Some European countries consider open drug scenes a “no go” area [4, 21]. In a recent study, just one-third of residents described their encounters with open drug scenes as negative/very negative [1]. Some areas in Berman, Germany, represent success in reducing drug scene-related nuisance by implementing tolerance zones, i.e., relocation of drug scenes from previous sites toward dedicated places with regard to more acceptance by the community and PWUDs [22]. Also, regarding the establishment of interventions such as drug consumption rooms (DCRs) that might lead to the improvement of public amenities, local residents mostly agree to establish them if these facilities can reduce the nuisance [12, 23]. In Iran, although, most residents felt frustrated from encountering PWUDs due to their poor conditions and maltreatment by police officers, they do not tolerate the presence of open drug scenes. They had concerns about public health and order issues. At the same time, they feel frustrated to observe people who cannot afford of their daily needs and forced to turn to disorder-based income generating activities.

Drug scenes contain stigma, and people going to hotspots, even without any visible drug problem, are considered problematic individuals that possibly affect perceived order concerns of local inhabitants. Some instances of perceived concerns of residents are unique to this study, such as fear of child abduction and fear of being attacked by PWUDs. Although participants complained of some issues as drug scene-related problems, we did not observe them. In addition to limited days and daytime of observing the drug scene-related problems in this study as well as conducting crackdown plans by the police during the study, this might be influenced by the level of tolerance of drug-related issues in Iran [1, 4, 24]. In other words, perceived fear in the neighborhood may indicate the resident’s perception of danger instead of the real one [24]. While the residents consider visible drug using/dealing in public as a warning sign that one needs to look after her/himself [25–27], using drugs in/around drug scenes might be due to PWUDs not having access to private space [26, 28–30]. In the current study, PWUDs who reside in drug scenes use fire due to the geographical position of Farahzad valley surrounded by trees and mountains [8]. The material configuration of the buildings that have windows toward drug scenes can bring smoke to nearby homes due to lighting fire by PWUDs.

Discarded paraphernalia in the neighborhood can induce a sense of inconvenience due to infected needles left in the environment that might result in a risk for infectious disease [17, 20]. Street fights and noise were other problems [2, 4, 25, 26]. In the current study, most of the fights possibly happened at night when residents need to fall sleep. In the research literature, garbage collection has been mentioned as a source of street-based income generating activity for PWUDs [31, 32], in our study, residents considered it as a source of neighborhood pollution, too.

As one would expect, according to the gender balance in the open drug scene activities [16], male drug users involve more in drug scene-related problems than women drug users [29]. There were some self-regulatory strategies by drug scene actors to hide inconvenient activities from the public view. For example, PWUDs avoided the most crowded areas to take drugs. They also did not use drugs during daytime. One of the main street-based income generation activities of PWUDs was garbage gathering as a more acceptable way in the community to obtain money for drugs. In addition, harm reduction innovative strategies such as digging a hole were implemented in the Farahzad valley to burry syringes. Despite implementing some self-regulatory strategies to reduce inconvenience in the neighborhood, the residents are unable tolerated drug scene activities.

Innovative harm reduction programs such as establishing peer-led networks in these areas are important to provide information and dialogue to local residents to make the risk and harm reduction policies more acceptable [33, 34]. Setting up a social reporting line and communication campaigns are also possibly tools in improving the level of social acceptance of PWUDs by citizens and reducing the perceived nuisance and concerns [35, 36].

## Limitations

This study, like others of its kind, features several limitations that should be noted. The study is cross-sectional, meaning the PWUDs and residents were questioned when the drug scene-related issues were still ongoing. While this was congruent with the research aims, the research design offers no information about the dynamic evolution of the phenomena under observation in terms of perceived problems. Our study has traditional limitations, such as problems with the generalization of findings to a broader population due to the small sample size. This study needs more robust research to detail concerns of PWUDs and other local inhabitants about practical issues of everyday coexistence with the drug scene-related situations. The observation of drug scene-related problems was limited to some days and during daytime due to the presence of watching guards who smugglers recruit to monitor and provide security for drug dealing in the drug scene. Although we could not observe some drug scene-related problems, possibly due to crackdown plans by the police, we observed manifestations of drug use in public, including drug-related litter. In addition, gender differences should be explored.

## Conclusion

Despite efforts of PWUDs to keep their activities invisible, drug scene-related issues are intolerable for neighbors. Residents and PWUDs have ambivalent attitudes. While they are concerned about the human rights of each other, drug scene-related activities have disturbed the neighbor’s daily life and economic activities. A cycle of drug scene-related issues reinforces and influences each other. In fact, disproportionate disciplinary strictures without meeting the daily needs of people involved in using and dealing drugs in public will typically not alleviate social problems in the neighborhoods. Besides law enforcement interventions in combination with innovative harm reduction programs should aim to improve coexistence of local society and PWUDs in a long-term planning approach.

## Data Availability

All data generated or analyzed during this study are included in this article. Further inquiries can be directed to the corresponding author.

## Abbreviations

PWUDs: People who use drugs
ODSs: Open drug scenes

## References

[1] Salkind NJ (2010). Encyclopedia of research design.

[2] Connoly J. Responding to open drug scenes and drug-related crime and public nuisance: towards a partnership approach: Council of Europe; 2006.

[3] Kammersgaard T (2020). Being ‘in place’, being ‘out of place’: Problematising marginalised drug users in two cities. Int J Drug Policy.

[4] Van Hout MC, Bingham T (2013). Open drug scenes and drug-related public nuisance: a visual rapid assessment research study in Dublin, Ireland. J Ethn Subst Abuse.

[5] Jalloh C, Illsley S, Wylie J, Migliardi P, West E, Stewart D (2017). What Goes Around: the process of building a community-based harm reduction research project. Harm Reduct J.

[6] Bless R, Korf DJ, Freeman M (1995). Open drug scenes: a cross-national comparison of concepts and urban strategies. Eur Addict Res.

[7] Zafarghandi MBS, Eshrati S, Vameghi M, Ranjbar H, Arezoomandan R, Clausen T (2019). Drug-related community issues and the required interventions in open drug scenes in Tehran, Iran: a qualitative study protocol. BMJ Open.

[8] Maarefvand M, Shirazi MS, Peyravi R, Farhoudian A (2017). Typology of street substance users' communities in Tehran, Iran. Addict Health.

[9] Grønnestad TE, Sagvaag H, Lalander P (2020). Interaction rituals in an open drug scene. Nordic Stud Alcohol Drugs.

[10] Waal H, Clausen T, Gjersing L, Gossop M (2014). Open drug scenes: responses of five European cities. BMC Public Health.

[11] Goffman E (2009). Relations in public.

[12] Grove RW (1988). An analysis of the constant comparative method. Int J Qual Stud Educ.

[13] Elo S, Kyngäs H (2008). The qualitative content analysis process. J Adv Nurs.

[14] Koch T (1994). Establishing rigour in qualitative research: the decision trail. J Adv Nurs.

[15] Fitzgerald JL, Threadgold T (2004). Fear of sense in the street heroin market. Int J Drug Policy.

[16] Zurhold H, Degkwitz P, Verthein U, Haasen C (2003). Drug consumption rooms in Hamburg, Germany: evaluation of the effects on harm reduction and the reduction of public nuisance. J Drug Issues.

[17] DeBeck K, Wood E, Qi J, Fu E, McArthur D, Montaner J (2011). Interest in low-threshold employment among people who inject illicit drugs: implications for street disorder. Int J Drug Policy.

[18] Cusick L (2007). Drug consumption rooms and regeneration: Environmental solutions to social problems. Int J Drug Policy.

[19] Schoenberger SF, Idrisov B, Sereda Y, Kiriazova T, Makarenko O, Bendiks S, et al. Police abuse and care engagement of people with HIV who inject drugs in Ukraine. Global Public Health. 2022:1–16. 10.1080/17441692.2022.2049341 PMC9515241 35343870

[20] DeBeck K (2010). Drug-related street disorder: evidence for public policy responses.

[21] Cusick L, Kimber J (2007). Public perceptions of public drug use in four UK urban sites. Int J Drug Policy.

[22] Prepeliczay S, Schmidt-Semisch H (2021). Tolerance zones: a pragmatic approach to respond to problems related to open alcohol and drug scenes in Bremen/Germany. Drugs Alcohol Today..

[23] Roth AM, Kral AH, Mitchell A, Mukherjee R, Davidson P, Lankenau SE (2019). Overdose prevention site acceptability among residents and businesses surrounding a proposed site in Philadelphia, USA. J Urban Health.

[24] Houborg E, Frank VA, Bjerge B (2014). From zero tolerance to non-enforcement: creating a new space for drug policing in Copenhagen, Denmark. Contemp Drug Probl.

[25] Lalander P (2020). Hooked on heroin: Drugs and drifters in a globalized world.

[26] Stevenson B (2010). Research into the nature and size of public drug injecting in Glasgow city.

[27] van der Poel A, Barendregt C, van de Mheen D (2003). Drug consumption rooms in Rotterdam: an explorative description. Eur Addict Res.

[28] DeBeck K, Wood E, Qi J, Fu E, McArthur D, Montaner J (2012). Socializing in an open drug scene: the relationship between access to private space and drug-related street disorder. Drug Alcohol Depend.

[29] Newcombe R. Multi-drug injecting in Manchester. A survey of 100 injecting drug users attending Lifeline Needle Exchange Scheme in 2006. 2007.

[30] Neale J, Tompkins C, Sheard L (2008). Barriers to accessing generic health and social care services: a qualitative study of injecting drug users. Health Soc Care Community.

[31] DeBeck K, Shannon K, Wood E, Li K, Montaner J, Kerr T (2007). Income generating activities of people who inject drugs. Drug Alcohol Depend.

[32] DeBeck K, Small W, Wood E, Li K, Montaner J, Kerr T (2009). Public injecting among a cohort of injecting drug users in Vancouver, Canada. J Epidemiol Community Health.

[33] Rafiey H, Alipour F, Moghanibashi-Mansourieh A, Mardani M (2019). The lived experiences of becoming a homeless person with addiction in Tehran: how they are withdrawn from the mainstream community?. J Soc Distress Homel.

[34] Mason K. Best practices in harm reduction peer projects. 2006.

[35] (EMCDDA) EMCfDaDA. Annual Report 2005: selected issue 1: Drug-related public nuisance—trends in policy and preventive measures. Luxembourg: Office for Official Publications of the European C0mmunities.

[36] Damon W, Callon C, Wiebe L, Small W, Kerr T, McNeil R (2017). Community-based participatory research in a heavily researched inner city neighbourhood: perspectives of people who use drugs on their experiences as peer researchers. Soc Sci Med.

